# In Vitro Comparative Study of Oxygen Plasma Treated Poly(Lactic–Co–Glycolic) (PLGA) Membranes and Supported Nanostructured Oxides for Guided Bone Regeneration Processes

**DOI:** 10.3390/ma11050752

**Published:** 2018-05-08

**Authors:** Daniel Torres-Lagares, Lizett Castellanos-Cosano, Maria-Angeles Serrera-Figallo, Carmen López-Santos, Angel Barranco, Agustín Rodríguez-González-Elipe, Jose-Luis Gutierrez-Perez

**Affiliations:** 1 Faculty of Dentistry, University of Seville, Avicena Street, 41009 Seville, Spain; lizettcastellanos@yahoo.es (L.C.-C.); maserrera@us.es (M.-A.S.-F.); jlgp@us.es (J.-L.G.-P.); 2 Institute of Materials Science of Seville (CSIC-University of Seville), Américo Vespucio Street nº 49, 41092 Seville, Spain; mclopez@icmse.csic.es (C.L.-S.); angel.barranco@csic.es (A.B.); arge@icmse.csic.es (A.R.-G.-E.)

**Keywords:** guided bone regeneration, polylactic, membranes, PLGA, oxygen plasma, nanomedicine

## Abstract

(1) Background: The use of physical barriers to prevent the invasion of gingival and connective tissue cells into bone cavities during the healing process is called guided bone regeneration. The objective of this in-vitro study was to compare the growth of human osteoblasts on Poly(Lactic–co–Glycolic) (PLGA) membranes modified with oxygen plasma and Hydroxyapatite (HA), silicon dioxide (SiO_2_), and titanium dioxide (TiO_2_) composite nanoparticles, respectively. (2) Methods: All the membranes received a common treatment with oxygen plasma and were subsequently treated with HA nanostructured coatings (n = 10), SiO_2_ (n = 10) and TiO_2_ (n = 10), respectively and a PLGA control membrane (n = 10). The assays were performed using the human osteoblast line MG-63 acquired from the Center for Scientific Instrumentation (CIC) from the University of Granada. The cell adhesion and the viability of the osteoblasts were analyzed by means of light-field microphotographs of each condition with the inverted microscope Axio Observer A1 (Carl Zeiss). For the determination of the mitochondrial energy balance, the MitoProbe™ JC-1 Assay Kit was employed. For the determination of cell growth and the morphology of adherent osteoblasts, two techniques were employed: staining with phalloidin-TRITC and staining with DAPI. (3) Results: The modified membranes that show osteoblasts with a morphology more similar to the control osteoblasts follow the order: PLGA/PO_2_/HA > PLGA/PO_2_/SiO_2_ > PLGA/PO_2_/TiO_2_ > PLGA (*p* < 0.05). When analysing the cell viability, a higher percentage of viable cells bound to the membranes was observed as follows: PLGA/PO_2_/SiO_2_ > PLGA/PO_2_/HA > PLGA/PO_2_/TiO_2_ > PLGA (*p* < 0.05), with a better energy balance of the cells adhered to the membranes PLGA/PO_2_/HA and PLGA/PO_2_/SiO_2_. (4) Conclusion: The membrane in which osteoblasts show characteristics more similar to the control osteoblasts is the PLGA/PO_2_/HA, followed by the PLGA/PO_2_/SiO_2_.

## 1. Introduction

Tissue engineering has been used as a strategy during the 21st century for the development of guided bone regeneration scaffolds and composites. In this manner, compared with other traditional methods, bone tissue engineering offers a new and interesting approach to bone repair. Guided bone regeneration consists of the placement of physical barriers that prevent the invasion of the connective or gingival tissue during the process of bone healing [1]. Among the physical barriers most commonly used in these biological processes is the Poly(Lactic–co–Glycolic) (PLGA) copolymer, due to previously described characteristics such as good bone adhesion, good vascularity, increased growth of osteoblastic cells on the surface of in vitro cultures, good biodegradability by hydrolysis, and subsequent elimination in the Krebs cycle in the form of CO_2_ and H_2_O [2]. In a previous work of our team, information about the characteristics and functions of scaffolds with biomedical applications was expanded, with special interest in scaffold construction using poly(lactic-co-glycolic acid) polymers [3].

Numerous published studies have focused on the in vitro and in vivo effect of modified PLGA by means of processes that allow its roughness to increase [4,5,6,7]. As it is well known, the internal cellular organization and its orientation are controlled by focal adhesions that mediate the regulating effects of the adhesion of the extracellular matrix. The distribution of the actin-myosin fibers depending on the properties of the surface is based on integrins that serve as mechanosensors, converting the mechanical signals of the medium into biological signals [4]. This scientific and biological evidence has allowed us to consider that the induction of the cellular activity of the osteoblasts can be further improved by modifying not only the roughness of the biomaterial surface, but also by the deposition of nanostructured coatings [5].

The objective of this in vitro study was to study which of the PLGA membranes coated with nanostructured hydroxyapatite (HA), silicon dioxide (SiO_2_), and Titanium Oxide (TiO_2_) presented a more optimal osteoblastic growth, in comparison with a control PLGA membrane.

## 2. Materials and Methods

For the experimental design of this in vitro study, four groups of scaffolds were analysed: PLGA control, PLGA-HA, PLGA-SiO_2_, and PLGA-TiO_2_, using the MG-63 human osteoblast line acquired at the Center of Scientific Instrumentation (CIC) at the University of Granada. This line of MG-63 shows a faster growth than the primary bone-forming lines but retains many of their characteristics, which makes it a good model in vitro. Firstly, a control of mycoplasma contamination was performed by PCR to verify that the cells were free of contamination. A method of detection by PCR (polymerase chain reaction) was used. The amplification of a band of approximately 500 bp was performed according to eight species of mycoplasma (*M. hyorhinis*, *M. arginini*, *M. pneumoniae*, *M. fermentans*, *M. orale*, *M. pirum*, *Acholeplasma laidlawii*, and *Spiroplasma mirum*) using a single pair of oligonucleotides corresponding to the 16S RNA. PCR was performed by taking an aliquot of the conditioned medium from the cells in culture after at least 48 h of culture [4,8,9]. MG-63 cells were cultured on control and experimental membranes.

Forty 50 µm thick resorbable inert PLGA scaffolds based on poly(lactic–co–glycolic) acid were fabricated using polycondensation (Institute of Materials Science, Seville, Spain). For the preparation of the membranes, 10 mL of a solution of PLGA (PLGA pellets with a copolymer ratio of 75:25 (lactic/glycolic acid) from Sigma-Aldrich Inc., St. Louis, MO, USA) in 1.5% dichloromethane was prepared by evaporation of the solvent on a Teflon plate for 48 h in air at room temperature, obtaining a film of a suitable consistency [4].

In the present research, the membranes’ surfaces of groups were exposed to pure oxygen plasma in a low pressure RF parallel plate reactor working at 13.56 MHz and 10 W for 1 min. The distance between the substrate and grid was 10 cm. This technique generates an etching effect, improving the surface roughness and favouring the adhesion of oxide layers to the PLGA substrates [10]. The treatment of PLGA substrates with oxygen plasma took place at close to ambient temperatures (RT: 23.0 ± 1.0 °C). It is important to stress that similarly to other plasma treatments reported in the bibliography, this plasma surface oxidation process did not affect the membrane structural integrity [10].

One group of membranes was coated with bioactive layers of SiO_2_ and another one with TiO_2_. The synthesis of the nanostructured oxide films was carried out at room temperature (RT: 23.0 ± 1.0 °C) by using hexametildisiloxane (HDMSO) or titanium tetrakis-isopropoxide (TTIP) as precursors. Total pressure during deposition was 4 × 10^−3^ Torr. Precursors were placed in a stainless steel receptacle through which oxygen was bubbled while heating at 305 K. The system consisted of an external microwave plasma source (SLAN, Plasma Consult, GMbh, Wuppertal, Germany) separated from the reactor chamber by a grounded grid to avoid the microwave heating of the PLGA substrates. Distance from the substrate and grid was 10 cm. The system was operated at 400 W with pure O_2_ as plasma gas [11,12,13,14].

Subsequently, the other membranes were coated with bioactive layers of HA by magnetron sputtering using a radiofrequency power of 50 W under an argon atmosphere at a pressure of 3 × 10^−3^ mbar [4,15]. Before deposition, the chamber was maintained at a base pressure of 1 × 10^−6^ mbar. The sputtering deposition was carried out using a copper coating that was 3 mm thick and 46 mm diameter white calcium phosphate. (HA) target (hydroxyapatite) was 4 mm thick, 46 mm in diameter, and 99.9% purity (Kurt J. Lesker Company, Jefferson Hills, PA, USA). The target sample distance was 10 cm and the deposition time was 40 min, obtaining a thickness of the coating of HA on PLGA of around 15 nm [4].

The membranes tended to double with temperature, so in order to avoid this phenomenon during the cultivation, a methodology was designed before carrying out the tests. Two dental adhesives were tested and their toxicity was analysed (Algasiv and Novafix) because there are contradictory studies on their biocompatibility in the literature [9]. A culture of the MG-63 cells was carried out on control wells with Algasiv and Novafix and the morphology of the adherent cells was visualized at 30 h. It was observed that Algasiv produced a morphological change of the cells, rounding them. However, Novafix adhesive did not produce morphological changes, so was selected. In addition, we also used a biocompatible plastic cylinder to press the membranes’ edges. In this combined manner, it was found that the membranes remained smooth and did not ripple during the in vitro study. Once the methodology of the culture was fine-tuned, the assays were developed.

**Test 1**: For the determination of cell adhesion and osteoblast viability, osteoblasts were cultured on the PLGA experimental (HA, SiO_2_, TiO_2_) and PLGA control membranes, in triplicate, plating 120,000 cells. At 24 h, the cultures were analysed by the microphotography of osteoblasts [16,17,18]. Previously, the cells had been fixed with 70% ethanol for 5 min, as it was not possible to observe them without prior fixation. Subsequently, the cells were stained with phalloidin-TRITC (Sigma Aldrich Quimica SL, Madrid, Spain) that marked F-actin fibers to observe a greater increase in cell morphology and prolongations. The cells adhered to the different surfaces were observed at 40× with the Axio Observer A1 inverted microscope (Carl Zeiss Iberia, S.L., Madrid, Spain). Analysis of the number of total cells adhered to the membranes and the percentage of viable cells by means of automatic cell counting (Cell Countess, Invitrogen Fisher Scientific–Spain, Madrid, Spain) by exclusion of the vital stain with trypan blue was performed [4].

**Test 2**: For the determination of the mitochondrial energy balance, the MitoProbe™ JC-1 Assay Kit (Fisher Scientific–Spain, Madrid, Spain) was employed. Osteoblasts were cultured on the PLGA experimental (HA, SiO_2_, TiO_2_) and PLGA control membranes, in triplicate, plating 120,000 cells. At 24 h, the cultures were analysed. JC-1 is a membrane permeable dye widely used for determining mitochondrial membrane potential in flow cytometry and fluorescent microscopy [19]. When mitochondria show good functioning, the probe accumulates in the mitochondria and forms aggregates, which are emitted as a red colour (~590 nm). When the mitochondrial membrane potential decreases during cellular damage phenomena, the emission of the fluorescence turns green (~529 nm), decreasing the red/green ratio, because of the passage to the monomeric form of the probe. The cells were observed at 20× with the Axio Observer A1 inverted microscope after a 30 min incubation (Carl Zeiss). Due to the structure of the SiO_2_ and TiO_2_ membranes, it was difficult to obtain conclusive results on the green fluorescence, since they had enough auto fluorescence at this wavelength. Therefore, the ratio could not be calculated with this approach. This is the reason why a quantification by flow cytometry was performed. The red and green fluorescence of the cells that adhered to the different membranes during 24 h were quantified by means of flow cytometry and stained with the JC-1 probe for 15 min, measuring the mitochondrial potential [4].

**Test 3**: For the determination of cell growth and the morphology of adherent osteoblasts, one staining process was employed. Osteoblasts were cultured on the PLGA experimental (HA, SiO_2_, TiO_2_) and PLGA control membranes, in triplicate, plating 120,000 cells. At 24 h, the cultures were analysed. This staining consisted of phalloidin-TRITC 50 μg/mL (red)—a fluorescent phallotoxin that can be used to identify filamentous actin (F-actin) [20]—along with the use of DAPI (40,6-diamidino-2-phenylindole) 30 μg/mL (blue)—a nuclear and chromosome counterstain emitting blue fluorescence upon binding to AT regions of DNA cells attached to the different membranes for 24 h [21]. Cells were fixed with 70% alcohol. Both techniques were visualised at 20× with the fluorescence microscope Axio Observer A1 (Carl Zeiss). Measurement of the size of the cells and comparison between the different membranes by quantification with ImageJ was performed. The measurement of the area of cells stained with phalloidin-TRITC was performed, measuring five representative cells from three different samples of each membrane, using the software ImageJ 1.48p (National Institutes of Health, 9000 Rockville Pike, Bethesda, Maryland, MD, USA) [4].

## 3. Results

### 3.1. Test 1

**Figure 1:**
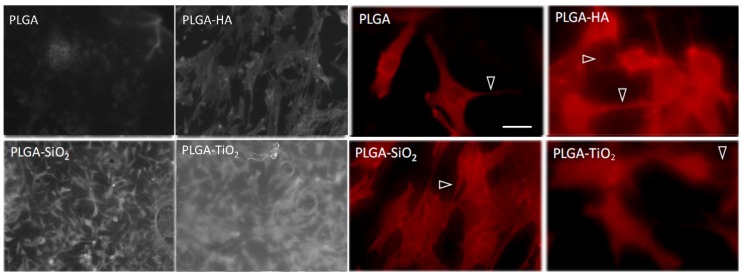
Microphotography of the osteoblasts on the membranes of PLGA, PLGA-HA, PLGA-SiO_2_, and PLGA-TiO_2_. Previously, the cells were fixed with 70% ethanol for five minutes, since without fixation it was not possible to observe them. Images taken at 5× with the fluorescence microscope Axio Observer A1 (Carl Zeiss). Bar in PLGA = 10 microns (same scale for all images). Filopodia are indicated with arrowheads.

**Figure 2:**
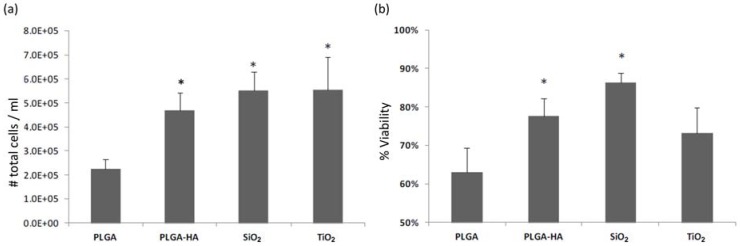
(**a**) Number of total cells adhered to the membranes. (**b**) percentage of viability of cells. *p < 0.05, statistically significant differences with respect to PLGA control membrane.

First, cell adhesion and osteoblast viability were determined. Osteoblasts attached to the PLGA membrane present a smaller size with a lower amount of adhered cells and fewer prolongations and filopodia. The osteoblasts bound to the PLGA-HA are larger and more similar to the control osteoblasts, with a large number of cells, extensions, and filopodia. The osteoblasts that are attached to the SiO_2_ are a little smaller and have many extensions and filopodia, although they are a little more fusiform. On TiO_2_, there is a high density of osteoblasts, but they are smaller and have fewer extensions than the osteoblasts adhered to PLGA-HA and SiO_2_. The osteoblasts that show a morphology more similar to that of the control are classified in the following order: PLGA-HA > SiO_2_ > TiO_2_ > PLGA (Figure 1). The results of the validated count observed in the microphotographs showed fewer cells adhered to the PLGA membrane. The viability was more compromised in this membrane, in addition to the one modified with TiO_2_. The percentage of viable cells bound to the membranes follows the following order: PLGA-SiO_2_ > PLGA-HA > PLGA-TiO_2_ > PLGA (Figure 2).

### 3.2. Test 2

**Figure 3:**
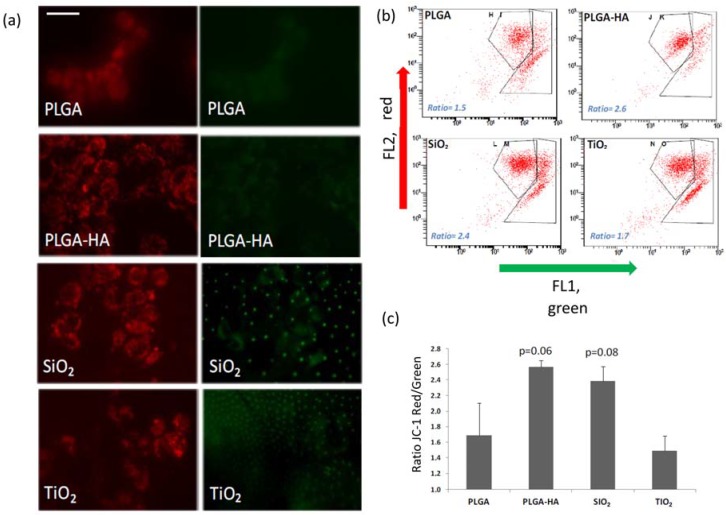
(**a**) Microphotography of the osteoblasts on studied membranes. (**b**) Representative histograms of the fluorescence visualization generated by the JC-1 probe by flow cytometry (FL2, red and FL1, green) on membranes. (**c**) The means + SD (standard deviation) of the ratio of the red/green fluorescence of the cells adhered to the membranes, 24 h after sowing and the value of the p-value of the Student t-test with respect to PLGA control membrane. Bar in PLGA = 10 microns (same scale for all images).

The results obtained after the determination of the mitochondrial energy balance, labelled with JC-1, are observed in Figure 3. A greater red/green ratio was observed for the osteoblasts adhered to the membranes PLGA-HA (2.57) and PLGA-SiO_2_ (2.38), than for PLGA (1.69) and PLGA-TiO_2_ (1.49), which indicates a better energy balance of the cells adhered to the membranes of PLGA-HA and SiO_2_ (Table 1).

### 3.3. Test 3

**Figure 4:**
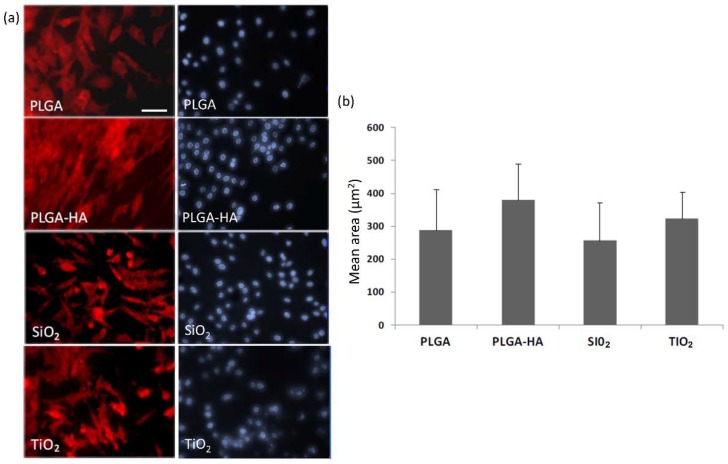
(**a**) Microphotography of the osteoblasts on studied membranes stained with phalloidin-TRITC 50 (red) and DAPI (blue). (**b**) The mean + SD of the areas of the cells adhered to the membranes is shown. Bar in PLGA = 10 microns (same scale for all images).

**Figure 5:**
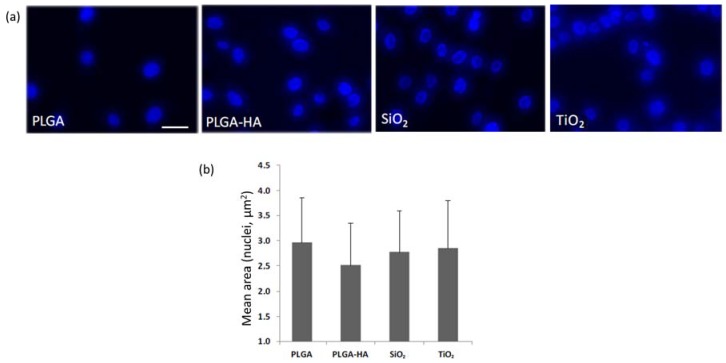
(**a**) Microphotography osteoblasts’ nucleus adhered on studied membranes. (**b**) The mean and standard deviation of the area of osteoblasts’ nucleus adhered on the membranes is shown. Bar in PLGA = 10 microns (same scale for all images).

The data observed in the determination of cell growth, and morphology of the adhered osteoblasts by visualizing the cultures with Phalloidin-TRITC (Sigma) and DAPI (Sigma), reinforce the conclusions indicated in test 1 regarding cellular morphology. The membrane where the cells show a greater area is in PLGA-HA (290 μm^2^), followed by TiO_2_ (320 μm^2^), PLGA (290 μm^2^), and finally SiO_2_ (260 μm^2^), although the differences did not reach significance between the membranes. The PLGA-HA membrane also has the maximum and minimum greater area between the measurements made on the membranes. The circularity of the cells in the membranes is similar to that of the control membrane, except for TiO_2_, in which the cells are more damaged (Figure 4). Additionally, a measurement was made of the size of the nuclei, which is also indicative of cell damage phenomena, such as death by apoptosis. The data obtained when measuring the area of the nuclei did not show a remarkable alteration (Figure 5).

## 4. Discussion

Synthetic biodegradable polyesters such as polyglycolic (PGA), polylactic (PLA), and their derivatives, for example, co-glycolic polylactic copolymer (PLGA), are being widely used in bone regeneration [22]. Oxygen plasma increases the rough surface of the membrane and its ability to stimulate bone regeneration (evaluated as the combination of bone neoformation, mineralization, resorption and presence of osteoclasts, and osteosynthesis activity) [23]. Previous studies in vitro and in vivo have compared the use of previously modified membranes with oxygen plasma and subsequently coated with oxide plasma films, finding that the functionalization of PLGA membranes with oxygen plasma improves the results in guided bone regeneration [4,5,6,7].

Within our team, we have carried out some previous studies that have been interested in these areas. The objective of the present article is the direct comparative study of a greater number of in vitro layers in order to provide data to other researchers if they wish to make progress in these layers. Our team has advanced to study in vivo in some of these configurations, which does not detract from the present study [4,5].

Although PLGA has been shown to have a good biocompatibility, PLGA polymers have been associated with hydrophobicity problems. This compromises its mechanical resistance and facilitates the release of acid waste by degradation, decreasing the pH and therefore favouring bacterial proliferation and the inflammatory response [24]. Inorganic materials such as tricalcium phosphate (TCP) or bioactive crystals have previously been used to modify the surface of the membranes and try to mitigate such disadvantages [22]. In this in vitro study, the treatment with oxygen plasma and the deposition of nanostructured silicon dioxide (SiO_2_), titanium dioxide (TiO_2_), and hydroxyapatite (HA) on membrane surfaces previously modified with oxygen plasma was evaluated.

In the present in vitro study, the modified membrane that showed the osteoblasts with a morphology more similar to the osteoblast control was the PLGA/PO_2_/HA membrane (*p* < 0.05). This result may be due to the fact that the HA used in this study is a synthetic material of the same composition as HA present in the human organism Ca_5_(PO_4_)_3_ (OH), with a great capacity to mimic the structure and composition of natural bone [25]. The synthetic HA (Ca_10_(PO_4_)_6_(OH)_2_) as a material for guided bone regeneration has a long history of use and its results are excellent [26]. Almost all materials or scaffolds used in guided bone regeneration base their composition on HA, since it is the main component of the mineralized connective tissue [27]. Torres-Lagares et al. [4] found in an in vitro and in vivo study that the incorporation of nanometric layers of HA in membranes of PLGA previously modified with oxygen plasma promotes greater osteosynthetic activity, new bone formation, and mineralization than the PLGA control group. As we have previously pointed out, these publications complement some of the configurations studied in this article with in vivo data, which does not detract from a comparative and direct study of different configurations that can be followed by other researchers.

In the present in vitro study, the membrane that showed the best cellular viability was that of PLGA/PO_2_/SiO_2_ (*p* < 0.05). It is interesting to note that no significant difference in cell size was found, although it was found in the number of cells that grew. This could indicate that the membranes are all suitable for cell growth, but that some allow a greater division of them to reach larger populations. This result may be due to the fact that the addition of silicon to alkaline phosphates can increase enzymatic activity and protein production in osteoblasts [28,29], producing a stimulating effect on cellular activity: proliferation, differentiation, and osteoblastic mineralization [30,31]. The membranes of PLGA/PO_2_/SiO_2_ and PLGA/PO_2_/HA presented a better energy balance of the cells adhered to the membranes.

In the present study, the PLGA/PO_2_/TiO_2_ membrane did not show better results than those membranes coated with HA and SiO_2_. These results coincide with those of a previous study conducted by Castillo-Dali et al. [5], in which membranes coated by PECVD with SiO_2_ and TiO_2_ that had been previously modified, either with or without oxygen plasma, were studied in vivo. The results found that incorporating layers of silicon dioxide into the PLGA membranes pretreated with oxygen plasma resulted in improved bone neoformation compared to the addition of TiO_2_ films.

This work only contributed the in vivo data of the experimentation of the experimental membrane, which is the reason why the data contributed in the present article complement the previously published data.

In relation to titanium dioxide, its main qualities are based on the ability of bone stimulation by reaction: they have an isoelectric point between 3.5 and 6.7 and low solubility [32]. This means that the surface is negatively charged at a physiological pH, which results in being able to react with biomolecules. The dielectric constant of TiO_2_ is comparable to that of water and, therefore, the molecularly charged interactions are similar to those of water [33]. The proteins that bind to TiO_2_ are formed by peptides and, in turn, by amino acids. These proteins act as specific ligands that genetically excite chemotaxis, proliferation, growth, and cell differentiation [34]. On the other hand, the adsorption of proteins in metals determines their good biocompatibility [35].

Related to the porosity of the membranes used, in a previous work [10], we reported that there is not evident weight loss of the PLGA sample after the oxygen plasma. Moreover, an in-depth GCIB-XPS study has proved that the oxygen plasma effect is able to reach only a few hundred nanometers, providing roughness enhancement and the generation of new functional groups. Plasma treatment changed the surface roughness from a value of root-mean square roughness (RMS) 0.34 nm for the control PLGA to 327 nm after the PO_2_ treatment. [10]

In general, the deposition techniques used do not alter the bulk properties of a polymer substrate and only alter their external surface. In detail, the SiO_2_ and HA thin film deposition processes do not induce changes in the surface roughness, presenting RMS values of 0.40 nm and 1.9 nm, respectively. Besides, the deposition of an SiO_2_ nanostructured coating on the PLGA membrane does not alter the composition and other characteristics of the organic membrane, as proved by previous FT-IR analyses [36]. No significant spectral changes were evidenced when comparing the PLGA membrane (50 μm) and the SiO_2_ films (15 nm), taking into the typical thickness proved by ATR analysis (tens of microns) [36].

The bioactivity, degradation behaviour, and osteoconductivity/osteoinductivity of calcium phosphate ceramics generally depend on the calcium/phosphate ratio, crystallinity, and phase composition [37]. The synthetic HA (Ca_10_(PO_4_)_6_(OH)_2_) shows good stability in the body, whereas tricalcium phosphates (α-TCP, β-TCP, Ca_3_(PO_4_)_2_) are more soluble. BCP (a mixture of HA and β-TCP) has intermediate properties depending on the weight ratio of stable/degradable phases. Therefore, the dissolution rate decreases in the following order: α-TCP > β-TCP > BCP > HA [37]. Due to their nature, Ca_3_(PO_4_)_2_ ceramics also exhibit high biocompatibility and the ability to bind with bone tissue under certain conditions; however, given their fragility, their clinical applications have been limited to non-carrier or low-load parts of the skeleton [38]. In fact, it is thought that nanoparticles of hydroxyapatite (nHA) are one of the most promising bone graft materials due to their ability to mimic the structure and composition of natural bone [25]. The HA used in this study is a synthetic material of the same composition as the HA present in the human organism Ca_5_(PO_4_)_3_ (OH). Synthetic HA as a material for GBR has a long history of use, and its results are excellent [26]. Almost all materials or scaffolds used in GBR base their composition on HA, the main component of mineralised connective tissue [27].

## 5. Conclusions

In this in vitro study, the membrane in which osteoblasts show characteristics of better cellular growth is PLGA-PO_2_-SiO_2_, followed by PLGA-PO_2_-HA. These in vitro data can be used by other researchers to decide upon which configuration to use in new experiences. Only some of these membranes need in vivo studies to complete our knowledge about them.

## Figures and Tables

**Table 1:** The means and standard deviation of the ratio of the red/green fluorescence on the cells adhered in the studied membranes are shown.

Ratio Red/Green	Measure 1	Measure 2	Measure 3	Mean	SD ^1^
PLGA	1.44	2.16	1.46	1.69	0.41
PLGA-HA	2.59	2.66	2.55	2.57	0.09
PLGA-SiO_2_	2.57	2.18	2.39	2.38	0.19
PLGA-TiO_2_	1.34	1.43	1.71	1.49	0.19

^1^ SD: Standard Deviation.

## References

[1] Hild N., Schneider O.D., Mohn D., Luechinger N.A., Koehler F.M., Hofmann S., Vetsch J.R., Thimm B.W., Müller R., Stark W.J. (2011). Two-layer membranes of calcium phosphate/collagen/PLGA nanofibres: In vitro biomineralisation and osteogenic differentiation of human mesenchymal stem cells. Nanoscale.

[2] Wang D.X., He Y., Bi L., Qu Z.H., Zou J.W., Pan Z., Fan J.J., Chen L., Dong X., Liu X.N. (2013). Enhancing the bioactivity of Poly(lactic-co-glycolic acid) scaffold with a nano-hydroxyapatite coating for the treatment of segmental bone defect in a rabbit model. Int. J. Nanomed..

[3] Castillo-Dalí G., Velázquez-Cayón R., Serrera-Figallo M.A., Rodríguez-González-Elipe A., Gutierrez-Pérez J.L., Torres-Lagares D. (2015). Importance of poly(lactic-co-glycolic acid) in scaffolds for guided bone regeneration: A focused review. J. Oral Implantol..

[4] Torres-Lagares D., Castellanos-Cosano L., Serrera-Figallo M.Á., García-García F.J., López-Santos C., Barranco A., Gonzalez-Elipe A.R., Rivera-Jiménez C., Gutiérrez-Pérez J.L. (2017). In vitro and in vivo study of poly(lactic–co–glycolic) (plga) membranes treated with oxygen plasma and coated with nanostructured hydroxyapatite ultrathin films for guided bone regeneration processes. Polymers.

[5] Castillo-Dalí G., Castillo-Oyagüe R., Terriza A., Saffar J.L., Batista A., Barranco A., Cabezas-Talavero J., Lynch C.D., Barouk B., Llorens A. (2014). In vivo comparative model of oxygen plasma and nanocomposite particles on PLGA membranes for guided bone regeneration processes to be applied in pre-prosthetic surgery: a pilot study. J. Dent..

[6] Castillo-Dalí G., Castillo-Oyagüe R., Batista-Cruzado A., López-Santos C., Rodríguez-González-Elipe A., Saffar J.L., Lynch C.D., Gutiérrez-Pérez J.L., Torres-Lagares D. (2017). Reliability of new poly (lactic-co-glycolic acid) membranes treated with oxygen plasma plus silicon dioxide layers for pre-prosthetic guided bone regeneration processes. Med. Oral Patol. Oral Cir. Bucal.

[7] Castillo-Dalí G., Castillo-Oyagüe R., Terriza A., Saffar J.L., Batista-Cruzado A., Lynch C.D., Sloan A.J., Gutiérrez-Pérez J.L., Torres-Lagares D. (2016). Pre-prosthetic use of poly(lactic-co-glycolic acid) membranes treated with oxygen plasma and TiO_2_ nanocomposite particles for guided bone regeneration processes. J. Dent..

[8] Wong-Lee J.G., Lovett M., Persing D.H., Smith T.F., Tenover F.C., White T.J. (1993). Rapid and sensitive PCR method for identification of Mycoplasma species in tissue culture. Diagnostic Molecular Microbiology: Principles and Applications.

[9] Paz-Pumpido F. (2005). Biocompatibilidad de los adhesivos dentinarios. Av. Odontoestomatol..

[10] Lopez Santos C., Terriza A., Portoles J., Yubero F., Gonzalez-Elipe A.R. (2015). Physiological Degradation Mechanisms of PLGA Membrane Films under Oxygen Plasma Treatment. J. Phys. Chem. C.

[11] Jacobs T., Declercq H., De Geyter N., Cornelissen R., Dubruel P., Leys C., Beaurain A., Payen E., Morent R. (2013). Plasma surface modification of polylactic acid to promote interaction with fibroblasts. J. Mater. Sci. Mater. Med..

[12] Borrás A., Barranco A., González-Elipe A.R. (2006). Design and control of porosity in oxide thin films grown by PECVD. J. Mater. Sci..

[13] Sánchez-Valencia J.R., Borrás A., Barranco A., Rico V.J., Espinós J.P., González-Elipe A.R. (2008). Preillumination of TiO_2_ and Ta_2_O_5_ photoactive thin films as a tool to tailor the synthesis of composite materials. Langmuir.

[14] Borrás A., Yanguas-Gil A., Barranco A., Cotrino J., González-Elipe A.R. (2007). Relationship between scaling behavior and porosity of plasma-deposited TiO_2_ thin films. Phys. Rev. B.

[15] Nieh T.G., Jankowski A.F., Koike J. (2001). Processing and characterization of hydroxyapatite coatings on titanium produced by magnetron sputtering. J. Mater. Res..

[16] Ruiz-Gaspa S., Nogues X., Enjuanes A., Monllau J.C., Blanch J., Carreras R., Mellibovsky L., Grinberg D., Balcells S., Díez-Perez A. (2007). Simvastatin and atorvastatin enhance gene expression of collagen type 1 and osteocalcin in primary human osteoblasts and mg-63 cultures. J. Cell. Biochem..

[17] Staehlke S., Rebl H., Finke B., Mueller P., Gruening M., Nebe J.B. (2018). Enhanced calcium ion mobilization in osteoblasts on amino group containing plasma polymer nanolayer. Cell Biosci..

[18] Di Toro R., Betti V., Spampinato S. (2014). Biocompatibility and integrin-mediated adhesion of human osteoblasts to poly(dl-lactide-co-glycolide) copolymers. Eur. J. Pharm. Sci..

[19] Huang L., Zhang Z., Lv W., Zhang M., Yang S., Yin L., Hong J., Han D., Chen C., Swarts S. (2013). Interleukin 11 protects bone marrow mitochondria from radiation damage. Adv. Exp. Med. Biol..

[20] Waggoner A., DeBiasio R., Conrad P., Bright G.R., Ernst L., Ryan K., Nederlof M., Taylor D. (1989). Multiple spectral parameter imaging. Methods Cell Biol..

[21] Kubista M., Akerman B., Nordén B. (1987). Characterization of interaction between DNA and 4′,6-diamidino-2-phenylindole by optical spectroscopy. Biochemistry.

[22] Jung R.E., Kokovic V., Jurisic M., Yaman D., Subramani K.F., Weber E. (2011). Guided bone regeneration with a synthetic biodegradable membrane: a comparative study in dogs. Clin. Oral. Implants Res..

[23] Shen H., Hu X., Yang F., Bei J., Wang S. (2007). Combining oxygen plasma treatment with anchorage of cationized gelatin for enhancing cell affinity of poly(lactide-co-glycolide). Biomaterials.

[24] Khang G., Jeon J.H., Lee J.W., Cho S.C., Lee H.B. (1997). Cell and platelet adhesions on plasma glow discharge-treated poly(lactide-co-glycolide). Biomed. Mater. Eng..

[25] Baino F., Novajra G., Vitale-Brovarone C. (2015). Bioceramics and Scaffolds: A Winning Combination for Tissue Engineering. Front. Bioeng. Biotechnol..

[26] Liu J., Kerns D.G. (2014). Mechanisms of guided bone regeneration: A review. Open Dent. J..

[27] Palmer L.C., Newcomb C.J., Kaltz S.R., Spoerke E.D., Stupp S.I. (2008). Biomimetic systems for hydroxyapatite mineralization inspired by bone and enamel. Chem. Rev..

[28] Botelho C.M., Brooks R.A., Best S.M., Lopes M.A., Santos J.D., Rushton N., Bonfield W. (2006). Human osteoblast response to silicon-substituted hydroxyapatite. J. Biomed. Mater. Res. A.

[29] Fielding G., Bose S. (2013). SiO_2_ and ZnO dopants in three-dimensionally printed tricalcium phosphate bone tissue engineering scaffolds enhance osteogenesis and angiogenesis in vivo. Acta Biomater..

[30] Obata A., Tokuda S., Kasuga T. (2009). Enhanced in vitro cell activity on silicon-doped vaterite/poly(lactic acid) composites. Acta Biomater..

[31] Pietak A.M., Reid J.W., Stott M.J., Sayer M. (2007). Silicon substitution in the calcium phosphate bioceramics. Biomaterials.

[32] Wieland M. (1999). Experimental Determination and Quantitative Evaluation of the Surface Composition and Topography of Medical Implant Surfaces and Their Influence on Osteoblastic Cell Surface Interactions. Ph.D. Thesis.

[33] Tiainen H., Wohlfahrt J.C., Verket A., Lyngstadaas S.P., Haugen H.J. (2012). Bone formation in TiO_2_ bone scaffolds in extraction sockets of minipigs. Acta Biomater..

[34] Ellingsen J.E., Thomsen P., Lyngstadaas S.P. (2006). Advances in dental implant materials and tissue regeneration. Periodontol. 2000.

[35] Länge K., Herold M., Scheideler L., Geis-Gerstorfer J., Wendel H.P., Gauglitz G. (2004). Investigation of initial pellicle formation on modified titanium dioxide (TiO_2_) surfaces by reflectometric interference spectroscopy (RIfS) in a model system. Dent. Mater..

[36] Terriza A., Vilches-Pérez J.I., de la Orden E., Yubero F., González-Caballero J.L., González-Elipe A.R., Vilches J., Salido M. (2014). Osteoconductive Potential of Barrier NanoSiO_2_ PLGA Membranes Functionalized by Plasma Enhanced Chemical Vapour Deposition. BioMed. Res. Int..

[37] Bose S., Tarafder S. (2012). Calcium phosphate ceramic systems in growth factor and drug delivery for bone tissue engineering: A review. Acta Biomater..

[38] Habraken W., Habibovic P., Epple M., Bohner M. (2016). Calcium phosphates in biomedical applications: Materials for the future?. Mater. Today.

